# Aneurysm of the Abdominal Aorta

**Published:** 1905-10-21

**Authors:** 


### ANEURYSM OF THE ABDOMINAL AORTA.

Professor Wm. Osler,[1] in a valuable paper, reviews his experience of aneurysms of the abdominal aorta as met with during a period of sixteen years at the Johns Hopkins Hospital. The cases were sixteen in number among an admission total of some 18,000 patients. The ratio of abdominal to thoracic aneurysm was about 1 to 10. Of the 16 patients 2 were females and 14 males. Nine patients were under forty years of age, and in three the disease had started before the thirtieth year. Only seven of the patients had been very heavy workers. Ten of the patients were alcoholics; in nine there was a definite, and in four others a doubtful, history of syphilis. In regard to symptoms the condition in one case is described as "latent" and was only discovered on post-mortem examination after death from pneumonia; in a second case there were "no symptoms," whilst in a third there were "no abdominal symptoms." In the remaining thirteen, pain of a persistent, often of an agonising character, was present. Pain was the most common, usually the earliest, and also the most prominent clinical feature of the series. In character it was generally "dull and boring," varied in some cases with paroxysms of frightful severity. Pressure on or stretching of nerves and erosion of the vertebræ were the responsible causes, though the latter condition was not always associated with pain. Rupture of the sac and the escape of blood into the retroperitoneal and muscular tissues is another cause of pain in abdominal aneurysm. The pain, it should be remembered, may simulate that of renal or hepatic colic or of appendicitis; it is sometimes referred to the distribution of the anterior crural or sciatic nerve. Of other symptoms in Dr. Osler's series nausea and vomiting were prominent in two cases; constipation was common; intermittent claudication was observed in one patient; in no instance was there hæmatemesis, and melæna was only noted in one of the cases, and that subsequent to operation by electrolysis and wiring. Thus, excepting pain, and where rupture occurred the features associated with that event, there were few symptoms and the patients were usually well-nourished and healthy-looking.

Of objective signs of abdominal aneurysm pulsation or throbbing is the most conspicuous, but it must be remembered that this may also be present in other circumstances. Even in health in most persons, more or less pulsation is visible between the ensiform cartilage and the navel when the recumbent posture is assumed. In spare subjects this pulsation may be especially appreciated in two areas —namely, first at the ensiform process or in the right costo-xiphoid angle, and, secondly, a little to the left of the navel; whilst between the two visible pulsation may or may not be present. After considerable exertion, and still more in the distention of the right heart as a consequence of valvular disease, pulsation in the epigastric region is very conspicuous; the actual impulse is due to the pushing down and out of the left lobe of the liver, though in occasional cases it directly depends upon a greatly dilated right ventricle. Again, visible aortic pulsation is met with in neurotic and hysterical states, especially in women. Signs of general debility often accompany this pulsation and in extreme cases there are inability to take food, pain, shortness of breath, and even attacks of hæmatemesis or melæna. A thrill may be present and also a soft systolic bruit, the latter even without pressure from the stethoscope. There is, however, no palpable expansile tumour and without this no bruit or thrill justifies a diagnosis of abdominal aneurysm.

A second association of preternatural pulsation in the upper abdomen is with the presence of tumours. Especially is this true of the large flat carcinoma of the stomach, and, indeed, of cancer of the stomach generally. Though the pulsation in such circumstances may be very forcible it is not expansile. The greatest difficulty in diagnosis exists in the case of comparatively small tumours situated directly over the course of the vessel, as here the throbbing is usually very pronounced and a bruit and thrill may also be present. In thin subjects the absence of an expansile character in the pulsation can usually be appreciated. Tumours of the pancreas, mesentery, and retroperitoneal glands may also lead to widespread pulsation in the upper abdomen. Again, in extreme anæmia the general marked throbbing of the arteries may reach its highest development in the abdominal aorta and here also there may be a thrill and bruit, whilst at the same time the pulse may have a "water-hammer" quality. In aortic insufficiency also the degree of abdominal pulsation may be sufficient to suggest aneurysm. The same is true of a sclerotic aorta in old men with thin abdominal walls. A very rare cause of abdominal pulsation is regurgitation along the inferior vena cava.

Another difficulty in the diagnosis of abdominal aneurysm is provided by those cases in which the sac has ruptured behind the peritoneum and has formed a large tumour in the upper abdomen or occupying one or both flanks and having little or no pulsation. Such a condition may be mistaken for a new growth and may lead to a proposal for surgical interference. The sudden development of pain and the presence of a non-pulsating tumour have on more than one occasion led in such cases to an abdominal incision on the view that the case was one of rapidly-growing sarcoma or other condition susceptible to operation. The rapid anæmia, emaciation, and slight fever which follow rupture tend to strengthen the suspicion that the case is one of new growth.

The treatment of abdominal aneurysm hardly offers a hopeful prospect. Occasionally Nature effects a cure, and Professor Osler mentions two instances of spontaneous healing. He has rarely found the rest and low diet treatment or the use of gelatine of much practical value. In the series of cases now described surgical measures were mainly adopted. In one case the abdomen was incised and an attempt made to compress the abdominal aorta, but the patient died on the table. In a second case, in which the tumour had so remarkable a degree of mobility that it was judged to affect one of the branches of the aorta rather than the main trunk itself an exploratory laparotomy was practised; this showed the aneurysm to belong to the aorta and it was so solid and the patient's general condition so good that nothing further was done. In seven of the cases the sac was wired and an electric current was passed. The results were as follows:—One patient died forty-eight hours after operation from rupture of the sac through the diaphragm into the pleural cavity. In a second case there was a fatal termination on the ninth day from hæmorrhage into the peritoneum; rupture of the sac on the fourteenth day was the cause of death in a third case. A fourth patient was much improved and was discharged at the end of four weeks; no subsequent history could be obtained. In a fifth case there was no change when the patient left the hospital some weeks later. The best result was in the sixth member of the series, a young man under thirty years of age when the evidences of aneurysm first developed. The operation was followed by reduction in the size of the sac, by cessation of the pain and complete relief of the nausea and vomiting—the latter having been specially troublesome symptoms. This patient was seen at intervals during three and a half years, at the end of which time he died from rupture of the sac. In the last case of the series hæmorrhage from the bowel followed the operation, but after this there was improvement. Later, however, rupture took place into the retroperitoneal tissues, and death occurred some six months after the operation.

[1] Lancet, Oct. 14, 1905.

